# Effect of Nurses’ Working Conditions Improvement Policy on Patient Outcomes in General Hospitals: A Quasiexperimental Study Using National Health Insurance Claims Data From Korea

**DOI:** 10.1155/jonm/4282547

**Published:** 2026-04-28

**Authors:** Hyo-Jeong Yoon, Hyunjeong Kwon

**Affiliations:** ^1^ Dankook University College of Nursing, Cheonan-si, Chungcheongnam-do, South Korea; ^2^ Keimyung University College of Nursing, Dalseo-gu, Daegu, South Korea, kmu.ac.kr

**Keywords:** difference-in-differences, nurses, nursing service insurance, nurse staffing, nurses’work environment, patient outcome, policy revision

## Abstract

**Background:**

A good nursing work environment is a key determinant of patient safety. In 2018, the Korean government introduced the Nurse Working Conditions Improvement Policy (NWCIP), accompanied by a revision of the nurse staffing grade calculation method. Under this policy, hospitals with revenue growth due to upward adjustments in nurse staffing grade were recommended to allocate revenue growth for improving nurses’ working conditions.

**Objective:**

To evaluate whether funding under the NWCIP, aimed at improving nurses’ working conditions, is associated with changes in patient outcomes.

**Methods:**

A quasiexperimental study was conducted using National Health Insurance claims data from 198,318 adult inpatients across 99 general hospitals. After confirming the parallel trends assumption, a difference‐in‐differences approach was used to evaluate the policy’s effect. Patient outcomes were compared between the first quarters of 2018 (baseline) and 2019 (postimplementation). Hospitals were categorized into an intervention group (those that received nurses’ working conditions improvement fund) and a comparison group (those that did not). Outcomes included in‐hospital mortality, 7‐day readmission, and length of stay. Among the 99 hospitals, 60 were assigned to the intervention group and 39 to the comparison group.

**Results:**

According to difference‐in‐differences analysis, the intervention group showed a 19% increase in 7‐day readmission (adjusted odds ratio = 1.19; 95% confidence interval: 1.09–1.29; *p* < 0.001) and a 5% decrease in length of stay (adjusted incidence rate ratio = 0.95; 95% confidence interval: 0.92–0.97; *p* < 0.001), with no significant change in in‐hospital mortality.

**Conclusions:**

General hospitals subject to the NWCIP did not show improved patient outcomes: They had a higher risk of readmission despite a shorter length of stay.

**Implications for Nursing Management:**

The use of revenue growth to improve nurses’ working conditions should be mandated rather than recommended, prioritizing high‐impact staffing investments, such as hiring additional nurses, most directly linked to patient outcomes.

## 1. Introduction

The nursing work environment is a critical factor influencing patient safety and quality of care. Studies have shown that a positive nursing work environment, as perceived by nurses, is associated with lower rates of patient falls, reduced risk of adverse patient outcomes, and decreased likelihood of missed nursing care [1–4]. In Korea, however, the nursing work environment has long been characterized by structural challenges. Compared to the United States and some European countries, nurses in Korea care for a substantially higher number of patients per nurse, resulting in excessive workloads [5, 6]. Pronounced wage disparities across regions and healthcare institutions have led to an uneven distribution of the nursing workforce, with a concentration of nurses in metropolitan areas and large tertiary hospitals [7–9]. Difficulties in achieving work–life balance, largely due to rigid shift systems and long working hours, have further exacerbated nurse turnover and workforce instability [9].

To address this issue, the Korean government introduced the Nurse Working Conditions Improvement Policy (NWICP) in the second quarter of 2018. The NWICP was implemented in conjunction with a revision of the Nursing Fee Differentiation Policy (NFDP), an incentive‐based reimbursement system in which inpatient nursing fees vary according to nurse staffing levels, with higher reimbursement provided to hospitals with better staffing (Supporting file 1) [10]. Following the revision of the NFDP calculation methods, some hospitals experienced upward adjustments in nurse staffing grades, with revenue growth. The NWICP targeted hospitals that experienced revenue growth recommended that at least 70% of the additional revenue be allocated to improving nurses’ working conditions.

Rather than constituting a mere technical adjustment to staffing grade due to revision in calculation methods, the NWCIP functions as a policy mechanism that channels financial gains from grade upward adjustments into structured reinvestment in nurses’ working conditions, thereby potentially contributing to improvements in patient outcomes. Accordingly, it is essential to examine the effects of the NWCIP on patient outcomes. Previous studies have elucidated early‐stage policy mechanisms, namely, nurse staffing grade upward adjustment derived from the revised calculation method and the magnitude of revenue growth [11, 12]. However, the association between NWCIP and patient outcomes has yet to be clearly established.

Therefore, we aimed to examine the effects of NWCIP on patient outcomes by comparing general hospitals that experienced revenue growth and allocated nurses’ working conditions improvement fund with those that did not. We assessed the effects of NWCIP on patient outcomes using the National Health Insurance (NHI) claims data from general hospitals.

## 2. Methods

### 2.1. Study Design

This quasiexperimental study used a difference‐in‐differences (DiD) design to evaluate the effects of the NWCIP on patient health outcomes in general hospitals.

#### 2.1.1. NWCIP

In this study, the policy evaluated was the NWCIP, which was introduced in 2018, allocating funds to improve nurses’ working conditions. Under South Korea’s universal NHI system, patients pay out‐of‐pocket copayments, while the remaining costs are reimbursed to healthcare institutions by the NHI following review [13]. Within this system, inpatient nursing services are reimbursed through an inpatient nursing fee, which under the NFDP was differentiated based on the nurse‐to‐bed ratio (see Supporting file 1). However, this bed‐based calculation method did not adequately reflect the actual number of hospitalized patients [14], and hospitals with low bed occupancy rates were systematically disadvantaged, constraining investments in improving the nursing work environment [14].

To address these limitations, the Korean government revised the NFDP calculation method, replacing the nurse‐to‐bed ratio with a nurse‐to‐patient census ratio, and introduced the NWCIP [10]. This policy was implemented for nontertiary hospitals that (1) are not located in urban areas (i.e., Seoul, metropolitan cities, or districts of Gyeonggi Province), (2) are public or quasipublic hospitals, or (3) are designated regional emergency medical centers. [10]. This targeted approach considered the concentration of Korean nurses in tertiary and metropolitan hospitals [15, 16] to minimize the risk of worsening regional imbalances in the nurse workforce. In addition, it supported the maintenance and strengthening of institutions responsible for essential care provision and public services.

The revision was applied uniformly once nurse staffing data were submitted, and hospitals could not opt in or out of the new calculation method. Therefore, treatment assignment was determined mechanically by the policy rather than by hospitals’ voluntary decision‐making. For example, when a hospital’s average daily inpatient census is lower than the number of beds, the revised calculation method can result in an upward adjustment of its nurse staffing grade (see examples in Supporting File 2). The NWCIP targeted those with an upward adjustment in nurse staffing grades, resulting in revenue growth from inpatient nursing fees, and recommended allocating at least 70% of the revenue growth to the nurses’ working conditions improvement fund. This nurses’ working condition improvement fund could be used for wage supplementation; hiring additional nurses; converting temporary or contract nurses to permanent positions; and indirect welfare measures, such as health screening, on‐site childcare operation, and educational support [10]. Therefore, the intervention in this study is operationalized as receipt of nurses’ working conditions improvement fund.

#### 2.1.2. Group Classification and Time Definition

##### 2.1.2.1. Time Definition

The NWCIP was implemented in the second quarter of 2018. Accordingly, the first quarter of 2018 was designated as the baseline period, with the postimplementation period defined as the first quarter of 2019. This design accounts for seasonal variation and minimizes the influence of common shocks. It avoids contamination from subsequent events, including the expansion of the NWCIP to metropolitan cities and districts of Gyeonggi Province in the fourth quarter of 2019 and the emergence of the COVID‐19 pandemic in the first quarter of 2020.

##### 2.1.2.2. Group Classification

General hospitals subject to the NWCIP were classified based on whether they received nurses’ working conditions improvement fund. Eligibility for the NWCIP was determined based on inclusion under the revised calculation method for nurse staffing grades. Among these, hospitals that experienced an upward adjustment in nurse staffing grade in the second quarter of 2018 and consequently received nurses’ working conditions improvement fund were categorized as the intervention group. By contrast, hospitals whose nurse staffing grades were unchanged from the second quarter of 2018 to the first quarter of 2019 and did not receive funds were categorized as the comparison group.

To ensure comparability and stable prepolicy conditions, the analysis was restricted to hospitals whose nurse staffing grades were unchanged during the four quarters preceding policy implementation. This restriction helped minimize potential spillover effects and establish a clear distinction between the intervention and comparison groups.

### 2.2. Data Sources

We used patient and hospital‐level data from the Health Insurance Review and Assessment Service (HIRA), which manages administrative claims data on behalf of the NHI. Anonymized claims data were collected for patients admitted to general wards of general hospitals between 2017 and 2019. These data enabled analysis of trends in patient outcomes during the four quarters prior to policy implementation to verify the parallel trends assumption required for DiD analysis. Postpolicy implementation effects were evaluated using data from the first quarter of 2019, which was 1 year after the policy was implemented in the second quarter of 2018. The dataset included patient demographics, clinical information (e.g., diagnoses, comorbidities, and death), NHI codes (e.g., inpatient nursing fees), and geographic location of the general hospital.

### 2.3. Study Population

**FIGURE 1:**
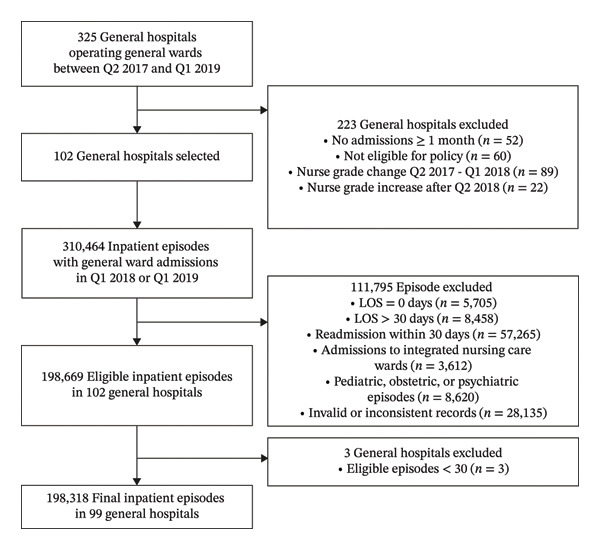
Flowchart of hospital and patient selection for the study population.

The study population was selected through a two‐stage process involving both hospital and inpatient episode‐level criteria, focusing on general hospitals subject to the NWCIP and their general ward inpatients (Figure 1).

At the hospital level, general hospitals were included if they operated general wards between the second quarter of 2017 and the first quarter of 2019 to assess parallel prepolicy trends and consistent outcome measurements. The exclusion criteria for hospitals were as follows: (1) no general ward inpatient admissions for at least 1 month during data collection; (2) ineligible for the NWCIP, including hospitals designated as tertiary hospitals or located in urban areas (i.e., Seoul, metropolitan cities, or districts of Gyeonggi Province) that were not designated as regional emergency medical centers; (3) changes in nurse staffing grade during the four quarters prior to policy implementation, to ensure stable prepolicy staffing conditions when grades were based on the bed‐to‐nurse ratio; (4) increase in nurse staffing grade after the second quarter of 2018, to ensure the intervention group reflected institutions that received fund immediately after the policy implementation and had sufficient time for potential outcomes to manifest in the postpolicy period. This also helped minimize spillover effects from staggered implementation; and (5) fewer than 30 eligible inpatient episodes during the baseline or postimplementation period. Of the eligible general hospitals, 99 were included in the final analysis, 60 in the intervention group and 39 in the comparison group.

At the inpatient episode level, episodes were included if admission occurred in general wards during the baseline or postimplementation period. Episodes were excluded if they met any of the following criteria: (1) length of stay (LOS) recorded as zero (i.e., same‐day admission and discharge) or exceeding 30 days, which is beyond the acute‐care range [17, 18]; (2) readmissions within 30 days of a prior discharge, retaining only the index admission to prevent duplication and clustering bias; (3) admissions to integrated nursing care wards, which were outside the scope of the policy; (4) admissions for pediatric, obstetric, or psychiatric care, which involve distinct nursing needs [19]; (5) invalid or inconsistent records (e.g., missing diagnosis codes and LOS discrepancies), including mismatches between LOS and inpatient billing codes due to administrative anomalies. A flowchart summarizing the inclusion and exclusion process is presented in Figure 1.

### 2.4. Patient Outcome Measures

Patient outcomes included in‐hospital mortality, 7‐day readmission, and LOS. In‐hospital mortality was defined as death during hospitalization. A 7‐day readmission was defined as readmission to an acute care hospital (hospitals, general hospitals, and tertiary hospitals) within 7 days of discharge after the index admission. The HIRA dataset used in this study covered all healthcare institutions nationwide, including acute care hospitals, enabling the identification of readmissions across institutions. To avoid misclassification of readmissions, cases in which patients died during the index admission or within 7 days after discharge at nonacute‐care facilities were excluded from the 7‐day readmission category. LOS was defined as the number of days from admission to discharge and was restricted to 1–30 days.

### 2.5. Covariate Measures

Both patient and hospital characteristics were included to adjust for covariates. Patient characteristics included age, sex, health insurance type (health insurance and medical aid), emergency admission, intensive care unit (ICU) admission, and the Charlson Comorbidity Index (CCI), which was calculated based on 17 comorbidities [20]. Hospital characteristics included location in the capital region (Seoul, Incheon, or Gyeonggi Province) and a capacity of 500 or more beds.

### 2.6. Statistical Analysis

The primary analysis employed a DiD approach to estimate the policy effect. To validate the parallel trends assumption, we examined prepolicy outcome trends using data from the four quarters preceding policy implementation. Specifically, we assessed graphical evidence based on box plots of group‐level outcomes aggregated by intervention status and quarter and estimated a group‐specific linear trend model as described by Wing et al. [21]. Visual inspection of the box plots showed no substantial differences in outcome trends between the intervention and comparison groups prior to policy implementation. In addition, the group‐by‐time interaction term was not statistically significant for any outcome, including in‐hospital mortality (*p* = 0.628), 7 day readmission (*p* = 0.599), and LOS (*p* = 0.241), indicating no evidence of differential prepolicy trends and supporting the application of the DiD design (Figure 2). Group‐level outcomes were calculated as unadjusted hospital‐level averages of patient‐level outcomes, aggregated by intervention status and quarter. Visual inspection of the corresponding box plots showed no notable differences between the groups prior to policy implementation.

FIGURE 2Prepolicy quarterly distributions of in‐hospital mortality, 7‐day readmission, and length of stay by intervention and comparison groups. Unadjusted prepolicy quarterly distributions of (a) in‐hospital mortality, (b) 7‐day readmission, and (c) length of stay, stratified by intervention and comparison groups. Box plots were constructed using hospital‐level average values based on patient‐level outcomes. The wide interquartile ranges reflect between‐hospital variation rather than within‐hospital patient‐level variation. The figure was created using SAS Enterprise Guide Version 7.1 within the HIRA customized remote data analysis system.

**Figure:**
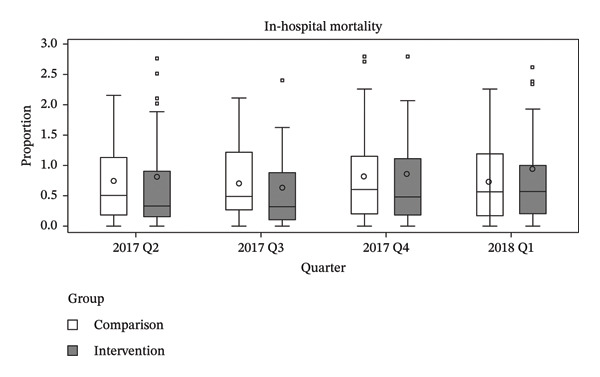
(a)

**Figure:**
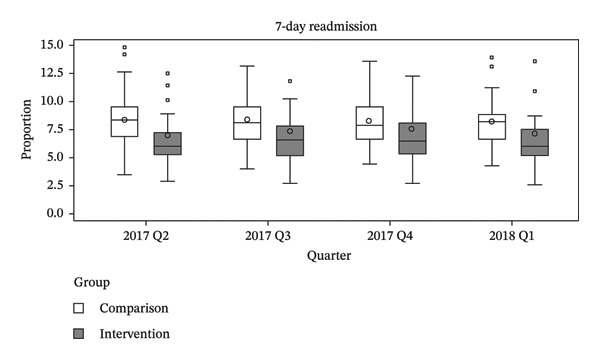
(b)

**Figure:**
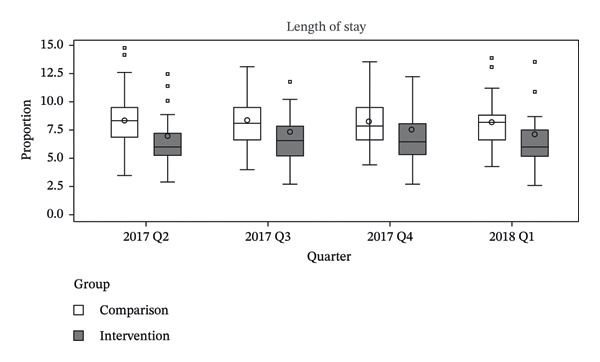
(c)

A group‐specific linear trend model tested whether the outcome trends over time differed between the intervention and comparison groups prior to policy implementation. We fitted a multilevel linear regression model of the form

(1) Yijt=α+β1Groupj+β2Timet+β3Groupj×Timet+uj+ϵijt,

• *Y*_*i* *j* *t*_ is the outcome for patient *i* in hospital *j* at time *t* (e.g., LOS, mortality, or readmission)
• Group_*j*_ is a binary indicator for the intervention group.
• Time_*t*_ is a continuous variable representing calendar quarters (coded −3, −2, −1, and 0).
• *u*_*j*_ is a random intercept for the hospital.
• *ϵ*_*i* *j* *t*_ is the patient‐level error term.

For all three outcome measures, the group‐by‐time interaction term (*β*_3_) was not statistically significant (all *p* > 0.05), indicating no evidence of differential prepolicy trends between the intervention and comparison groups and supporting the parallel trends assumption.

To estimate the effect of the policy on patient outcomes, a DiD framework was applied using data from the baseline and postimplementation periods. The primary outcomes were in‐hospital mortality (binary), 7‐day acute care readmission after discharge (binary), and LOS (continuous count variable). We used multilevel models with random intercepts for hospitals to account for patient clustering within hospitals. For mortality and readmission, multilevel logistic regression was performed; results were reported as adjusted odds ratios (aORs). For LOS, multilevel zero‐truncated negative binomial regression was performed; results were expressed as adjusted incidence rate ratios (aIRRs). The DiD specification included fixed effects for group, time, and group × time interaction, which captured differential changes in outcomes attributable to the policy.

(2) Yijt=α+δTreatPostjt+γGroupj+τPostt+Xijtβ+uj+ϵijt,

• TreatPost_*j* *t*_ = 1 for intervention group hospitals in the postimplementation period.
• X_*i* *j* *t*_ is a vector of covariates at the patient and hospital levels.
• *δ* estimates the policy effect (DiD estimator).

All statistical analyses were performed using SAS Enterprise Guide Version 7.1 within the HIRA customized remote data analysis system.

### 2.7. Ethical Considerations

The present study was approved as exempt by the Institutional Review Board of Seoul National University, and informed consent was waived by the Ethics Committee as the HIRA database pseudonymized patient data for research purposes (IRB No. E2308/001‐013).

## 3. Results

We analyzed 105,055 inpatient episodes from 99 hospitals (68,352 from intervention hospitals and 36,703 from comparison hospitals) at baseline and 93,263 inpatient episodes (58,799 from intervention hospitals and 34,464 from comparison hospitals) postimplementation. Among the 60 hospitals in the intervention group and the 39 hospitals in the comparison group, a higher proportion of intervention hospitals were located in the capital area (Seoul, Incheon, and Gyeonggi Province) or had 500 beds or more. In the comparison group, nurse staffing grades were unchanged from before to after the policy, with Grade 7 (lowest grade) being the most common, followed by Grade 6 and then Grade 1 (highest grade). By contrast, because hospitals in the intervention group received funds under the policy, they experienced upward adjustments in nurse staffing grades. Before policy implementation, Grade 3 was the most common, followed by Grades 2 and 6. After policy implementation, Grade 2 became the most prevalent, followed by Grade 1 and then Grade 3. No hospitals in the intervention group had Grade 1 staffing at baseline. Hospital characteristics in the comparison group remained largely unchanged between the pre‐ and postimplementation periods, consistent with the absence of policy exposure (Table 1).

**TABLE 1:** Hospital characteristics by intervention status and period.

Category		Baseline		Postimplementation	
		Comparison	Intervention	Comparison	Intervention
Location	Capital area	6 (15.4)	17 (28.3)	6 (15.4)	17 (28.3)
	Noncapital area	33 (84.6)	43 (71.7)	33 (84.6)	43 (71.7)

Size	≥ 500 beds	5 (12.8)	10 (16.7)	5 (12.8)	10 (16.7)
	< 500 beds	34 (87.2)	50 (83.3)	34 (87.2)	50 (83.3)

Nurse grade	Grade 1	5 (12.8)	—	5 (12.8)	17 (28.3)
	Grade 2	2 (5.1)	14 (23.3)	2 (5.1)	18 (30.0)
	Grade 3	2 (5.1)	18 (30.0)	2 (5.1)	12 (20.0)
	Grade 4	1 (2.6)	9 (15.0)	1 (2.6)	5 (8.3)
	Grade 5	—	3 (5.0)	—	2 (3.3)
	Grade 6	8 (20.5)	10 (16.7)	8 (20.5)	5 (8.3)
	Grade 7	21 (53.8)	6 (10.0)	21 (53.8)	1 (1.7)

Patients admitted to intervention hospitals were younger, with a higher proportion of those aged under 65 years and a lower proportion aged 75 years or older, compared to patients in comparison hospitals. The proportion of patients covered by medical aid was lower in intervention hospitals than in comparison hospitals. By contrast, a higher proportion of patients in intervention hospitals were admitted through the emergency department or received care in the ICU; their CCI scores were also higher.

In‐hospital mortality and 7‐day readmission were consistently higher in the comparison group than in the intervention group, both before and after policy implementation. For the analysis of 7‐day readmissions, 1439 patients who died within 7 days of discharge without being readmitted to an acute‐care hospital were excluded. Average LOS was more than one day longer in the comparison group than in the intervention group (Table 2).

**TABLE 2:** Patient characteristics and outcome incidences by intervention status and period.

Category		Baseline		Postimplementation	
		Comparison	Intervention	Comparison	Intervention
Age	< 65	20,936 (57.0)	41,848 (61.2)	19,831 (57.5)	36,474 (62.0)
	65≤ age < 75	6615 (18.0)	12,147 (17.8)	6305 (18.3)	10,775 (18.3)
	75∼	9152 (24.9)	14,357 (21.0)	8328 (24.2)	11,550 (19.6)

Sex	Male	17,521 (47.7)	32,650 (47.8)	16,672 (48.4)	28,515 (48.5)
	Female	19,182 (52.3)	35,702 (52.2)	17,792 (51.6)	30,284 (51.5)

Health insurance type	Health insurance	33,391 (91.0)	63,236 (92.5)	31,337 (90.9)	54,410 (92.5)
	Medical aid	3312 (9.0)	5116 (7.5)	3127 (9.1)	4389 (7.5)

Emergency admission	Yes	9359 (25.5)	19,177 (28.1)	8596 (24.9)	14,791 (25.2)
	No	27,344 (74.5)	49,175 (71.9)	25,868 (75.1)	44,008 (74.8)

ICU admission	Yes	1346 (3.7)	3389 (5.0)	1379 (4.0)	2730 (4.6)
	No	35,357 (96.3)	64,963 (95.0)	33,085 (96.0)	56,069 (95.4)

CCI		1.26 ± 1.58	1.35 ± 1.67	1.22 ± 1.57	1.30 ± 1.67
Comorbidities	Myocardial infarction	272 (0.7)	903 (1.3)	266 (0.8)	796 (1.4)
	Congestive heart failure	2096 (5.7)	4957 (7.3)	1952 (5.7)	4537 (7.7)
	Peripheral vascular disease	1011 (2.8)	2126 (3.1)	1042 (3.0)	2039 (3.5)
	Cerebrovascular disease	2688 (7.3)	6934 (10.1)	2654 (7.7)	6466 (11.0)
	Dementia	1228 (3.3)	2000 (2.9)	1114 (3.2)	1692 (2.9)
	Chronic pulmonary disease	7133 (19.4)	11,396 (16.7)	5643 (16.4)	8880 (15.1)
	Ulcer disease	3051 (8.3)	5974 (8.7)	2701 (7.8)	4119 (7.0)
	Mild liver disease	5536 (15.1)	13,846 (20.3)	5184 (15.0)	12,307 (20.9)
	Diabetes	7692 (21.0)	16,016 (23.4)	7169 (20.8)	13,146 (22.4)
	Diabetes with complications	1545 (4.2)	3364 (4.9)	1413 (4.1)	2237 (3.8)
	Hemiplegia or paraplegia	332 (0.9)	798 (1.2)	310 (0.9)	582 (1.0)
	Moderate or severe renal disease	924 (2.5)	2411 (3.5)	884 (2.6)	2178 (3.7)
	Any malignancy without metastasis	3135 (8.5)	4572 (6.7)	2749 (8.0)	4004 (6.8)
	Metastatic solid tumor	446 (1.2)	609 (0.9)	414 (1.2)	526 (0.9)

In‐hospital mortality cases		284 (0.8)	443 (0.6)	230 (0.7)	376 (0.6)
7‐day readmission cases		2860 (7.9)	4203 (6.2)	2471 (7.2)	3691 (6.3)
Length of stay		7.27 ± 6.40	6.21 ± 5.78	7.15 ± 6.36	6.01 ± 5.78

*Note:* Comorbidities with a prevalence of less than 1% were included in the statistical analysis but were excluded from this table. These include connective tissue disease, moderate or severe liver disease, and acquired immune deficiency syndrome.

Abbreviations: CCI = Charlson Comorbidity Index; ICU = intensive care unit.

As described in the Methods section, there was no evidence of differential prepolicy trends between the intervention and comparison groups. Multivariable regression models accounting for patient clustering within hospitals were used to estimate adjusted differences in outcomes between the intervention and comparison groups before and after policy implementation. At baseline, differences in in‐hospital mortality or LOS were nonsignificant between the intervention and comparison groups. The proportion of patients readmitted within 7 days was significantly lower in the intervention group (aOR: 0.86, 95% confidence interval [CI]: 0.74–0.99, *p* = 0.042). Following policy implementation, the overall proportion of 7‐day readmissions decreased by 7% (aOR: 0.93, 95% CI: 0.88–0.98, *p* = 0.009) and LOS decreased by 2% (aIRR: 0.98, 95% CI: 0.96–0.995, *p* = 0.012). Changes in in‐hospital mortality were nonsignificant. Interaction terms between the intervention group and the postimplementation period indicated a 19% increase in 7‐day readmissions (aOR: 1.19, 95% CI: 1.09–1.29, *p* < 0.001) and a 5% decrease in LOS (aIRR: 0.95, 95% CI: 0.92–0.97, *p* < 0.001), compared to the comparison group. No statistically significant differences in in‐hospital mortality were observed between the groups (Table 3).

**TABLE 3:** Adjusted ORs and IRRs indicating the differences in patient outcomes.

	**In-hospital mortality**		**7-day readmission**		**Length of stay**	
	**aOR (95% CI)**	**p** **value**	**aOR (95% CI)**	**p** **value**	**aIRR (95% CI)**	**p** **value**

Intervention vs. comparison group at baseline	1.09 (0.67–1.75)	0.735	0.86 (0.74–0.99)	0.042	0.85 (0.68–1.05)	0.131
Postimplementation vs. baseline in all hospitals	0.84 (0.70–1.00)	0.055	0.93 (0.88–0.98)	0.009	0.98 (0.96–0.995)	0.012
Intervention × postimplementation interaction	1.08 (0.80–1.46)	0.602	1.19 (1.09–1.29)	< 0.001	0.95 (0.92–0.97)	< 0.001

*Note:* All models adjusted for patient characteristics (sex, age, health insurance type, emergency admission, intensive care unit admission, and the Charlson Comorbidity Index) and hospital characteristics (location and size).

Abbreviations: aIRR = adjusted incidence rate ratio; aOR = adjusted odds ratio; CI = confidence interval.

## 4. Discussion

In this study, we examined whether the NWCIP, which encourages general hospitals to use revenue growth from inpatient nursing fees, was associated with changes in patient outcomes. 60.6% of the general hospitals were classified as intervention hospitals that received funding under the NWCIP following an upward adjustment in nurse staffing grades. This proportion is comparable to findings from a previous nationwide study, which reported that 62.0% of the 255 general hospitals were eligible for the nurses’ working conditions improvement fund [5]. To our knowledge, this is the first study to examine the impact of the NWCIP on patient outcomes and provide insights into policy implications.

We assumed that the NWCIP would improve the nurses’ working conditions and enhance the quality of nursing care, ultimately leading to better patient outcomes. Contrary to our expectations, intervention hospitals showed a pattern of shorter LOS coupled with higher 7‐day readmission risk. No association was observed with in‐hospital mortality. Although a shorter LOS may be interpreted as improved treatment efficiency, it should be interpreted in conjunction with other patient outcomes, such as readmissions. Evidence on the relationship between LOS and readmission is mixed. Some studies have reported that longer LOS is associated with a higher likelihood of readmission [22, 23]. Others have suggested the opposite; for example, in patients with acute myocardial infarction, longer LOS was negatively associated with readmission [24], suggesting that adequate treatment and stabilization before discharge are important for preventing readmissions. Early discharge strategies designed to shorten LOS have raised concerns about patient safety: premature discharge was identified as a potential driver of readmissions [25]. Consistently, Chen et al. [26] have reported an increased risk of readmission in patients discharged early. In this context, the pattern observed in our study, shorter LOS with higher odds of readmission, may indicate no improvement in patient safety and could reflect early discharge or insufficient discharge planning and transitional care. Although our analysis did not directly examine the causal relationship between LOS and readmission, we cannot rule out the possibility that hospitals pursued greater efficiency while discharge management processes were not sufficiently strengthened, thereby increasing readmission risk.

One possible explanation for the negative outcome in readmission in the intervention group is the presence of structural differences between the intervention and comparison groups. Specifically, the revised calculation methods may operate more favorably in hospitals with a lower average inpatient census and lower and more variable bed occupancy, such that grade upward adjustment and the associated revenue growth can occur and be maintained in the absence of a meaningful decrease in the number of patients per nurse, which is directly linked to nursing workload [12]. Another explanation is that the NFDP relies on quarterly averages of inpatient censuses and nurse counts, which may fail to capture short‐term nurse‐to‐patient ratio fluctuations. Even when the number of nurses per shift is constant, substantial day‐to‐day variations in patient volume can result in fluctuations in effective nurse staffing levels. Studies have suggested that greater variability in daily nurse staffing is associated with lower quality of nursing care and worse patient outcomes [27]. This may help explain why intervention hospitals did not translate into improved patient outcomes in our study.

Another reason is that, due to limitations in the available data, we classified hospitals based on whether nurses’ working conditions improvement funds were generated rather than on the actual extent to which the funds were used to improve nurses’ working conditions. Furthermore, because the use of the nurses’ working conditions improvement fund was recommended rather than mandated, the actual allocation of funds toward improving nurses’ working conditions may have varied across hospitals. According to Cho et al. [12], a one‐level upward adjustment in nurse staffing grade is associated with an estimated nurses’ working conditions improvement fund of 1.44–7.26 million Korean won per nurse (equivalent to 996–5030 USD), representing a substantial potential investment. Although hospitals were encouraged to allocate at least 70% of the additional revenue growth to nurses’ working conditions improvement fund and were required to submit expenditure reports for monitoring, as of Q4 2018, only 269 of 365 hospitals that experienced revenue growth have submitted the documentation [28]. Among general hospitals, only 55 of 128 reported spending at least 70% of revenue growth toward the nurses’ working conditions improvement fund, such as hiring additional nurses [28]. A recent review reported that the organizational financial return on nursing investment is limited and contradictory [29], potentially weakening hospitals’ motivation to translate revenue growth into substantive staffing investments or workload reduction. Taken together, these findings suggest that formal allocation alone is insufficient to ensure the effective use of revenue growth, underscoring the need for stronger accountability and monitoring mechanisms.

The impact of revenue growth allocated to the nurses’ working conditions improvement fund on patient outcomes may differ by spending category, reflecting time lags between investment and observable effects. For example, direct labor investments, particularly hiring of additional nurses, can reduce nurses’ workload and increase nursing hours per patient day, and thus may be more likely to yield comparatively instant improvements in patient outcomes [30, 31]. By contrast, indirect working condition improvement investments, such as on‐site childcare or education support, are more likely to exert their effects on patient outcomes over the long term. Studies have suggested that a higher proportion of well‐educated nurses and more experienced nurses is associated with better patient outcomes, including lower mortality and reduced readmission [32–34]. Investments that support workforce development and retention may therefore be necessary, but their benefits may not be immediate because they require time for nurses to accumulate clinical experience and translate educational support into enhanced competence. Accordingly, if the nurses’ working conditions improvement fund was primarily allocated to such indirect working condition improvements, the absence of observable changes in patient outcomes in the short term would be unsurprising.

### 4.1. Limitations

Our study has several limitations. First, in our study, we could not confirm how much and for what purposes general hospitals used the allocated nurses’ working conditions improvement fund, as detailed expenditure information was unavailable. Consequently, we could not determine whether or how the nurses’ working conditions improvement fund translated into meaningful changes in nurse staffing level, workload, and working conditions at the hospital level. Furthermore, although we sought to isolate the effect of the policy‐driven grade adjustment, limited administrative data (e.g., lack of nurse counts) prevented us from distinguishing whether grade changes reflected actual staffing increases or the revised calculation method. To mitigate this, we restricted the sample to hospitals with stable grades in the four quarters before the intervention and defined groups based on grade changes in Q2 2018; nevertheless, we could not directly verify hospital‐level changes in nurse‐to‐patient ratios. This limits the interpretability of the estimated policy effects. Second, as our study aimed to minimize potential external effects of the COVID‐19 pandemic on patient outcomes, we measured postimplementation outcomes only in 2019, the prepandemic period. This limits our study to capturing short‐term effects, and long‐term effects could not be assessed. Third, hospital information was deidentified; therefore, we were unable to determine whether hospitals were established under national legislation, one of the eligibility criteria for the NWCIP. As a result, this criterion could not be incorporated into sample selection and should be considered when interpreting findings. Fourth, hospitals in the intervention group that were classified as Grade 1 before the second quarter of 2018 were excluded, as it was not feasible to assess the effects of the nurse staffing grade adjustment resulting from the revision of the NFDP’s staffing calculation method. Lastly, regarding the study period, our estimates may be affected by contemporaneous external shocks, such as the cumulative effects of nursing school enrollment expansions in the 2010s, which potentially increased nurse supply. Therefore, the generalizability of the findings may be limited, and these considerations should be considered when interpreting the results.

## 5. Conclusions

In this study, we evaluated the association between the NWCIP and patient outcomes. The policy was intended to use revenue growth generated through upward adjustment in nurse staffing grades to improve nurses’ working conditions, including through increased staffing. However, we found that general hospitals subject to the NWCIP did not demonstrate improved patient outcomes. To better achieve the policy’s intended objectives, enhancing nurse staffing and improving working conditions, it may be necessary not only to revise the current recommendation into a mandate requiring allocation of revenue growth to the nurses’ working conditions improvement fund but also to require verified spending and formal reporting of its use, with enforcement mechanisms (e.g., recoupment of funds) for noncompliance.

### 5.1. Implications

#### 5.1.1. Policy Implication

Our findings suggest that improvements in patient outcomes may depend not only on allocating revenue growth to nurses’ working conditions improvement fund but also on how these funds are implemented and monitored. Accordingly, nurses’ working conditions improvement fund should be mandated for use in improving nurses’ working conditions, supported by clear spending rules and stronger accountability mechanisms. In addition, a phased allocation strategy is warranted, prioritizing direct staffing investments (e.g., hiring additional nurses) in the short term, while expanding support for workforce development and retention over the long term to better link this policy to sustained improvements in patient outcomes. Furthermore, we should assess whether the nurses’ working conditions improvement fund improved nurses’ perceived working conditions or environment using a structured, validated instrument (e.g., the PES‐NWI), which is widely used in nursing practice environment research, including Magnet‐related studies [35].

However, without clear evidence that investing in nurses increases hospitals’ financial returns, financial incentives alone may be insufficient to drive sustained workforce investment [29]. Given that nurse‐to‐patient ratios and nursing care time are key determinants of patient outcomes [36], stronger legal requirements, such as the mandated minimum staffing ratios of California, may be needed to ensure adequate staffing and protect patient safety. Before moving toward full legal mandates, the NFDP reporting system, which is based on 3‐month averages of patient census and nurse staffing, should be revised to better reflect the actual timing of nursing service delivery to incorporate patient severity, because studies on inpatient nursing fee have suggested that severity adjustment requires shorter measurement intervals (e.g., monthly or weekly) rather than quarterly averages [37, 38].

#### 5.1.2. Organizational or Managerial Implications

Studies have suggested that nurse‐led councils lead to increased communication and shared decision‐making around staffing and resource allocation, improving the perception of the nursing work environment [39]. In the context of the NWCIP, establishing nurse‐led councils focused on the use of the nurses’ working conditions improvement fund, which may help ensure that investments reflect the needs and priorities of frontline nurses. Such councils can serve as a practical governance mechanism by identifying priorities and monitoring whether the funds translate into meaningful improvements at the point of care.

## Author Contributions

Hyo‐Jeong Yoon contributed to conceptualization, methodology, formal analysis, data curation, writing the original draft, and reviewing and editing the manuscript. Hyunjeong Kwon contributed to methodology, writing the original draft, and reviewing and editing the manuscript.

## Funding

This research received no specific grant from any funding agency in the public, commercial, or non‐profit sectors.

## Conflicts of Interest

The authors declare no conflicts of interest.

## Supporting Information

Additional supporting information can be found online in the Supporting Information section. *Supporting Information*

## Supporting information


**Supporting Information 1** Supporting file 1: Nursing fee differentiation policy general ward in Korea (JNM).

**Supporting Information 2** Supporting file 2: Illustrative example of nurse staffing grade changes after the revised NFDP nurse staffing calculation method and calculation of revenue growth and the nurse work condition improvement fund in the grade‐change group.

## Data Availability

The data used in this study were accessible to researchers only via remote access during the analysis period. The raw data are owned by the Health Insurance Review and Assessment Service (HIRA) and cannot be shared.
