# Supplementary material for: Effect of Nurses’ Working Conditions Improvement Policy on Patient Outcomes in General Hospitals: A Quasiexperimental Study Using National Health Insurance Claims Data From Korea
Source: J Nurs Manag. 2026 Apr 28;2026:4282547. doi: 10.1155/jonm/4282547 (PMC13125350; doi:10.1155/jonm/4282547)
Supplement: Supplementary file 1 — Supporting Information 1 Supporting file 1: Nursing fee differentiation policy general ward in Korea (JNM). [file JONM-2026-4282547-s001.docx]

**Supplementary file 1. Nursing fee differentiation policy general ward in Korea**

| **Hospital type** | **Gr** | **Calculation method** | | **Ratio** | **Nursing fee differentiation** | **Relative index** | **Nursing fee**  **(KRW)** |
| --- | --- | --- | --- | --- | --- | --- | --- |
|  |  | **Mar.2018** | **Apr. 2018** |  |  |  |  |
| General hospital  located in a rural area (not in Seoul, metropolitan cities, districts of Gyeonggi province) or designated as a regional emergency medical centers | 1 | **Bed**:nurse^a^ | **Patient**: nurse^b^ | < 2.5 : 1 | 110% of grade 2 inpatient fee | 1.68 | 61,320 |
|  | 2 |  |  | ≥ 2.5 : 1 and < 3.0 : 1 | 110% of grade 3 inpatient fee | 1.53 | 55,750 |
|  | 3 |  |  | ≥ 3.0 : 1 and < 3.5 : 1 | 115% of grade 4 inpatient fee | 1.39 | 50,680 |
|  | 4 |  |  | ≥ 3.5 : 1 and < 4.0 : 1 | 110% of grade 5 inpatient fee | 1.21 | 44,070 |
|  | 5 |  |  | ≥ 4.0 : 1 and < 4.5 : 1 | 110% of basic inpatient fee | 1.10 | 40,060 |
|  | **6** |  |  | **≥ 4.5 : 1 and < 6.0 : 1** | **Basic inpatient nursing fee** | **1.00** | **36,420** |
|  | 7 |  |  | ≥ 6.0 : 1 | 0~5% reduction from basic fee  or same as basic inpatient fee^c^ | 0.95  ~1.00 | 34,600  ~36,420 |

Source: Health Insurance Review & Assessment Service (HIRA), February 2018; Fee Schedule File (Full Version), effective January 1, 2018.

^a^ The number of beds refers to the average number of beds in the previous quarter, and the number of nurses refers to the average number of nurses employed during the previous quarter, rather than the number of nurses actually working per shift.

^b^ The number of beds refers to the average number of beds in the previous quarter, and the number of nurses refers to the average number of nurses employed during

^c^ For grade 7, a 5% reduction is applied if the hospital is located in Seoul or six metropolitan cities (Busan, Ulsan, Daejeon, Daegu, Incheon, Gwangju), and a 2% reduction is applied if it is located in other regions. However, medical institutions located in medically underserved areas are exempt from this reduction even if they are classified as Grade 7.
