# Supplementary material for: Effect of Nurses’ Working Conditions Improvement Policy on Patient Outcomes in General Hospitals: A Quasiexperimental Study Using National Health Insurance Claims Data From Korea
Source: J Nurs Manag. 2026 Apr 28;2026:4282547. doi: 10.1155/jonm/4282547 (PMC13125350; doi:10.1155/jonm/4282547)
Supplement: Supplementary file 2 — Supporting Information 2 Supporting file 2: Illustrative example of nurse staffing grade changes after the revised NFDP nurse staffing calculation method and calculation of revenue growth and the nurse work condition improvement fund in the grade‐change group. [file JONM-2026-4282547-s002.docx]

**Supplementary file 2. Illustrative example of nurse staffing grade changes after the revised NFDP nurse staffing calculation method, and calculation of revenue growth and the nurse work condition improvement fund in the grade-change group**

| **Group** | **Nurse**^a^  **(A)** | **Bed**^b^  **(B)** | **Average**  **daily patients census**^c^  **(C)** | **Nurse staffing grade** | | **Grade diff** | **Nursing fee difference** | **Nursing fee revenue growth** | **Nurses’ working conditions improvement fund** |
| --- | --- | --- | --- | --- | --- | --- | --- | --- | --- |
|  |  |  |  | **Bed:nurse**  **(D=B:A)** | **Patient:nurse**  **(E=C:A)** | **F=E-D** | **G** | **H=GXCX(the number of days)** | **I=HX0.7** |
| Comparison | 40 | 100 | 100 | Grade 2  (2.5:1)  =(100/40:1) | Grade 2  (2.5:1)  =(100/40:1) | 0 | 0 | None | None |
| Intervention | 40 | 100 | 80 | Grade 2  (2.5:1)  =(100/40:1) | Grade 1  (2.0:1)  =(80/40:1) | - 1 | 61,320-55,750  =KRW 5,570  (approximately USD $4) | KRW 41,292,000  (USD 29,000)  =(5,570) X80patients  X93days | KRW 28,904,400  (USD 20,000)  =41,292,000X0.7 |

**Note.** The additional inpatient nursing fee was calculated in case of quarter (3 months) had 93 days.

^a^ The number of nurses was defined as the average number of nurses employed over a three-month period. For example, if the numbers of nurses employed in July, August, and September were 42, 40, and 38, respectively, the average number of nurses for the quarter was calculated as (42 + 40 + 38) / 3 = 40.

^b^ The number of beds was defined as the average number of licensed beds over the quarter (three-month period).

^c^ The average daily patient census was calculated using a midnight (00:00) census. For each day, the inpatient count was obtained by adding daily admissions to, and subtracting daily discharges from, the number of inpatients present at midnight. Admissions lasting less than six hours and not generating inpatient charges were excluded from the inpatient count. The average daily patient census was then calculated as the sum of daily inpatient counts over three months divided by the total number of days in that period. For example, if the cumulative number of daily inpatients over 83 days was 6,640, the average daily patient census was calculated as 6,640 ÷ 83, resulting in an average of 80 patients per day.
